# Leptin as a critical regulator of hepatocellular carcinoma development through modulation of human telomerase reverse transcriptase

**DOI:** 10.1186/1471-2407-10-442

**Published:** 2010-08-19

**Authors:** Nikolaos Stefanou, Vassilis Papanikolaou, Yoichi Furukawa, Yusuke Nakamura, Aspasia Tsezou

**Affiliations:** 1 University of Thessaly, Medical School, Department of Biology, Larissa, Greece; 2 Institute of Biomedical Research and Technology, Larissa, Greece; 3 University of Tokyo, Institute of Medical Science, Division of Clinical Genome Research, Tokyo, Japan; 4 University of Tokyo, Institute of Medical Science, Laboratory of Molecular Medicine, Tokyo, Japan; 5 University of Thessaly, Medical School, Laboratory of Cytogenetics and Molecular Genetics, University Hospital of Larissa, Larissa, Greece

## Abstract

**Background:**

Numerous epidemiological studies have documented that obesity is associated with hepatocellular carcinoma (HCC). The aim of this study was to investigate the biological actions regulated by leptin, the obesity biomarker molecule, and its receptors in HCC and the correlation between leptin and human telomerase reverse transcriptase (hTERT), a known mediator of cellular immortalization.

**Methods:**

We investigated the relationship between leptin, leptin receptors and hTERT mRNA expression in HCC and healthy liver tissue samples. In HepG2 cells, chromatin immunoprecipitation assay was used to study signal transducer and activator of transcription-3 (STAT3) and myc/mad/max transcription factors downstream of leptin which could be responsible for hTERT regulation. Flow cytometry was used for evaluation of cell cycle modifications and MMP1, 9 and 13 expression after treatment of HepG2 cells with leptin. Blocking of leptin's expression was achieved using siRNA against leptin and transfection with liposomes.

**Results:**

We showed, for the first time, that leptin's expression is highly correlated with hTERT expression levels in HCC liver tissues. We also demonstrated in HepG2 cells that leptin-induced up-regulation of hTERT and TA was mediated through binding of STAT3 and Myc/Max/Mad network proteins on *hTERT *promoter. We also found that leptin could affect hepatocellular carcinoma progression and invasion through its interaction with cytokines and matrix mettaloproteinases (MMPs) in the tumorigenic microenvironment. Furthermore, we showed that histone modification contributes to leptin's gene regulation in HCC.

**Conclusions:**

We propose that leptin is a key regulator of the malignant properties of hepatocellular carcinoma cells through modulation of hTERT, a critical player of oncogenesis.

## Background

Obesity is an important risk factor for many types of cancer, including hepatocellular carcinoma (HCC) [1,2]. Among adipocytokines, that are the main body weight regulators, leptin, the 16-KDa nonglycosylated protein product of the Ob gene, has a central role [3,4]. It is a multifunctional peptide hormone with a wide range of biological activities including neuroendocrine function [5], angiogenesis [6,7], bone formation [8] and modulation of immune responses [9,10]. Leptin exerts its actions through its six isoforms of receptors, which are membrane spanning glycoproteins with cytoplasmic domains of varying length [11].

Leptin's signaling is thought to be transmitted mainly by the Janus-activated Kinase/signal transducers and activators of transcription (JAK/STAT) pathway [12]. Of the seven human STAT genes, STAT3 has been shown to be activated in a wide variety of human tumors and tumor cell lines and its activation is accompanied by increased expression of important cell cycle and survival regulators, such as cyclin D1, c-myc and survivin [13,14]. Many STAT3 target genes are key components of the regulation of cell cycle progression from G1 to S phase [15].

At present, a biological explanation for the association between obesity and HCC is not known. It seems that there is a strong relationship between adipocytokines, such as leptin, and HCC but the molecular mechanisms have not been clarified yet. Hepatocarcinogenesis is a multi-step process involving different genetic alterations that ultimately lead to malignant transformation of the hepatocyte [16,17]. One of the molecular events that underlie the multigenetic process of hepatocarcinogenesis is activation of human telomerase reverse transcriptase (hTERT)/telomerase which is normally suppressed in most human somatic tissues after birth [18,19].

In the present study we investigated, for the first time, the relationship between leptin, leptin receptors and hTERT mRNA expression in HCC. We also attempted to elucidate on the molecular pathways that may mediate this interaction by investigating the regulation of *hTERT *gene promoter by histone acetylation status as well as STAT3 and c-myc transcription factors. Finally, the biological effects of leptin in HCC progression through inflammatory cytokines such as IL-1, IL-6, TGF and MMPs were assessed.

## Methods

### Subjects

The study protocol conformed to the ethical guidelines of the 1975 Declaration of Helsinki as reflected in a priori approval by the local Ethical Committee of the University Hospital of Larissa and by the Institutional Review Board (Institute of Medical Science, University of Tokyo). Specifically, control liver tissue specimens were obtained after oral informed consent from 23 patients (eleven male, twelve female; mean age 54.9 years, range 37-84 years) during an operation that was performed for cholelithiasis (cholecystectomy). All these individuals had apparently no evidence of chronic liver disease and normal ALT (alanine aminotransferase) values (26.6 ± 4.9 U/L), tested negative for HBsAg, anti-HCV and anti-HIV antibodies and denied ever having used hepatotoxic drugs, herbals, or having abused alcohol or injected drugs.

Twenty three liver tissue samples from HCC patients were used in this study, which were purchased from Biomax (US Biomax Inc, MD, USA) and were also provided from the University of Tokyo (thirteen male, ten female; mean age: 58.4 years; range: 45-75 years). Written informed consent was obtained from the patients. The diagnostic criteria for HCC were based on the conclusions of the Barcelona-2000 EASL conference while the histological diagnosis was made according to the AJCC/UICC classification system [20,21]. From the 23 HCC tissue samples, 8 were due to HBV-related cirrhosis and 15 were due to HCV-related cirrhosis.

### Cell cultures, reagents and treatments

HepG2 hepatocellular carcinoma cells were used and were cultured in RPMI 1640 medium (Gibco, Paisley, Scotland, UK) supplemented with 10% fetal bovine serum (Gibco, Paisley, Scotland, UK), L-Glutamine 2 mM (Gibco, Paisley, Scotland, UK), penicillin 100 IU/ml and streptomycin 100 μg/ml (Gibco, Paisley, Scotland, UK), at 37°C in 5% CO_2_. After 16 hours of serum starvation, the culture media were changed to serum free media containing leptin. Cultures were treated with human recombinant leptin at 25, 50, 100, 200 ng/ml (R&D Systems, Minneapolis, MN, USA). Cell culture supernates were removed, centrifuged and stored at -80°C until assayed. Leptin, TGF-b1, IL-6, IL-1b and IL-1a were measured using commercially available assays according to manufacturers' instructions (R&D Systems, Minneapolis, MN, USA).

### RNA isolation and Real-time PCR

Each sample was homogenized and total cellular RNA was extracted, reverse transcribed to cDNA and real-time PCR was performed for leptin, OB-Rs, OB-Rl and telomerase, as previously described [22,23]

### Immunohistochemistry for hTERT, leptin and OB-R

Immunohistochemical staining for hTERT and leptin expression was completed using antihuman hTERT antibody (PC563) (EMD Biosciences, Merck KGaA, Darmstadt, Germany), A20 leptin polyclonal Ab (pAb) (Santa Cruz Biotechnology, Santa Cruz, USA), or the M18 ObR pAb, (Santa Cruz Biotechnology) according to standard IHC procedures [24].

### Cell viability

Cell viability was determined with the MTT assay using the TACS MTT kit (R&D Systems, Minneapolis, MN, USA) according to manufacturer's instructions. HepG2 proliferation was assessed in the presence of increasing concentrations of leptin (0-200 ng/ml) or in the absence of leptin (siRNA treatment against leptin). Cell proliferation was examined at 12h, 24h and 48h after addition of leptin.

### TRAP assay

TRAP (telomeric repeat protocol assay) assay was performed using the TeloTAGGG telomerase PCR ELISA PLUS kit (Roche, Indianapolis, IN, USA) as previously described [25].

### Small interfering RNA treatment

HepG2 cells were transfected with dsRNA oligonucleotides for leptin using Lipofectamine 2000 reagent (Invitrogen, Carlsbad, CA). Different doses of siRNAs were administered at first for either 24, 48, 72 hours, in order to define the optimum dosage and time for a satisfying silencing, controlled by real time RT-PCR and ELISA (cell culture supernates). Negative controls (scrambled) were used in order to verify the absence of toxicity for the different doses administered.

### Chromatin immunoprecipitation

Chromatin Immunoprecipitation was performed using a ChIP assay kit (Upstate USA, Inc., Charlottesville, VA, USA). The immunoprecipitated DNAs were amplified by PCR with the primers indicated below. For leptin promoter (proximal promoter, forward: 5'-CCCTCTAACCCTGGGCTTC-3'; reverse: 5'-ACTATGGCGCAAGGACCAG-3'), for hTERT promoter (set 1 for STAT3, forward: 5'-CCAAACCTGTGGACAGAACC-3'; reverse: 5'-AGACTGACTGCCTCCATCGT-3', set 2 for STAT3, forward: 5'- GGGGTGTCTTCTGGGTATCA-3'; reverse: 5'-AAGGGCTGTGTTTGTGAATTG-3', proximal hTERT promoter, forward: 5'- TGCCCCTTCACCTTCCAGCTC-3'; reverse: 5'- GTGGCCGGGGCCAGGGCTT-3').

### Flow cytometry

Cell cycle distribution was determined by flow cytometry. At least 10.000 events were collected for each sample. Intracellular staining antibodies against MMP-1, MMP-9, proMMP-13 were used for cytometric analysis of HepG2 cells according to manufacturers instructions (R&D Systems, Minneapolis, MN, USA). Effect of leptin treatment (50, 200 ng/ml for 48 h and 100 ng/ml for 2 months) and leptin siRNA on MMP-1, MMP-9 and MMP-13 protein levels were evaluated.

### Statistical analysis

Statistical analysis was performed as previously described [22].

## Results

### Leptin, OB-Rl and OB-Rs expression in liver tissues of HCC patients

**Figure 1:**
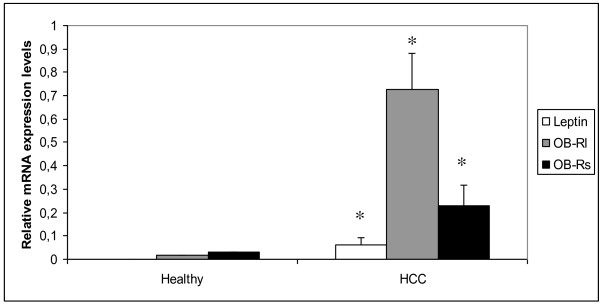
**Leptin, OB-Rl or OB-Rs expression levels in healthy and HCC liver tissues**. Comparison of liver tissues of healthy individuals and HCC patients with respect to mean leptin, OB-Rl, OB-Rs expression levels (leptin or OB-Rl or OB-Rs mRNA copies/PBGD copies) obtained after real time RT-PCR analysis. Bars, means ± standard deviation,*, p < 0.05 compared to healthy liver tissues.

In order to test the malignant dynamics of leptin in liver, we evaluated leptin and leptin receptors mRNA and protein expression using real-time RT-PCR and immunohistochemistry (IHC) respectively, in HCC and non-HCC liver tissues. Leptin was not expressed in any healthy liver tissue, but was expressed in 18 out of 23 HCC tissues as evaluated by RT-PCR or IHC (78.2%). More specifically, regarding real-time PCR data, mean leptin levels were 6.1 ± 3.21 × 10ˉ², while no difference in leptin expression levels was found between the HBV and HCV subgroups of the HCC group. Significant differences were observed between the mean OB-Rl and OB-Rs mRNA levels in HCC liver tissues (0.726 ± 0.155 and 0.227 ± 0.092, respectively,) and healthy tissues (0.0165 ± 0.0031 and 0.0292 ± 0.00194, respectively) (p < 0.001) (Figure 1).

### Correlation of leptin expression with hTERT expression

Interestingly, taking into account our previous findings in chronic viral hepatitis and HCC (altered leptin and hTERT mRNA levels in HCC or chronic viral hepatitis liver samples compared to healthy liver samples), we proceeded to determine whether there is an association between leptin and hTERT mRNA expression [22,23]. We found a significant association between leptin and hTERT mRNA expression only in HCC livers (r = 0.79, p < 0.05).

### Leptin affects hTERT expression levels and TA in HCC cells

**Figure 2:**
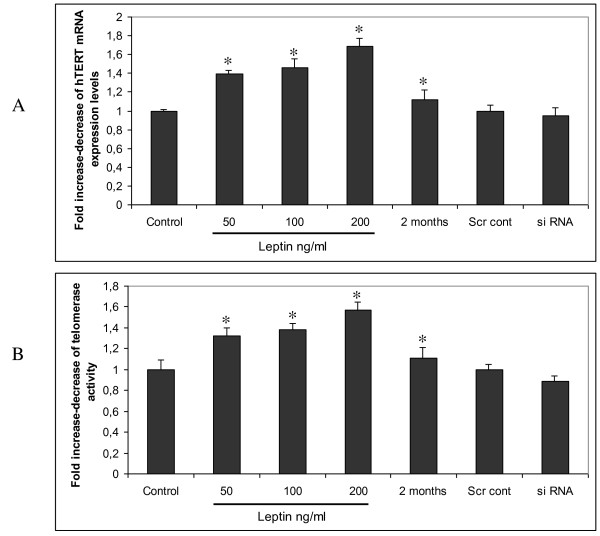
**Leptin promotes hTERT expression and TA in HepG2 cells**. (a) Total RNA was isolated and its expression was evaluated using real-time RT-PCR, to determine changes in the level of hTERT mRNA expression after normalization to PBGD expression. All data were presented as a fold induction relative to untreated cells. Columns, mean of three independent experiments done in triplicate; bars, SD; *, p < 0.05 compared to untreated cells, (b) Telomerase activity after leptin and leptin siRNA treatment as mentioned above.

The association between leptin and hTERT/TA in HCC samples prompted us to study the effect of leptin administration on hTERT in HepG2 cells. When HepG2 cells were treated with leptin concentrations of 50, 100, 200 ng/ml for 48 hours and 100 ng/ml for 2 months, we observed that hTERT mRNA levels and TA were significantly increased (Figure 2a, b). We then blocked leptin's expression in HepG2 cells using siRNA against leptin and transfection with liposomes and did not observe a significant decrease in hTERT mRNA levels and TA (Figure 2a, b).

### The JAK/STAT3 pathway and the Myc/Max/Mad network are important for leptin-mediated up-regulation of hTERT expression

**Figure 3:**
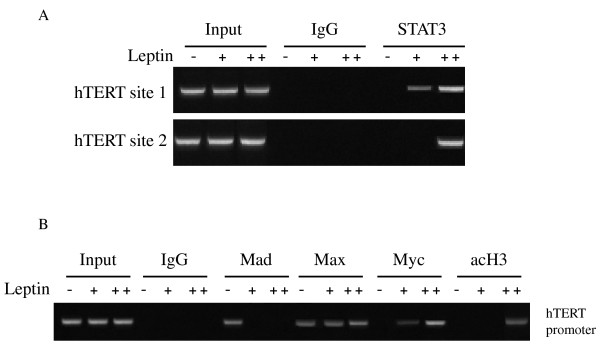
**Leptin enhances the binding of STAT3, Myc, H3 to *hTERT *promoter**. (a) ChIP assay was performed using anti-STAT3 antibody or irrelevant anti-immunoglobin G antibody as negative control. Input samples are total genomic DNAs used as control for the PCR. PCR primers covering the STAT3 binding sites of *hTERT *promoter region were used to detect promoter fragment in immunoprecipitates. Leptin (-), untreated cells, Leptin (+), 200 ng/ml for 48 h, Leptin (++), 100 ng/ml for 2 months. (b) ChiP assay was performed using anti-Mad1, anti-Max, anti-Myc and anti-acetylated H3 antibodies or irrelevant anti-IgG antibody as negative control. PCR primers covering proximal hTERT promoter were used. Leptin (-), untreated cells, Leptin (+), 200 ng/ml for 48 h, Leptin (++), 100 ng/ml for 2 months

To gain insight into the mechanism underlying the leptin-mediated transactivation of hTERT promoter on HCC cells, we next examined signal transduction pathways possibly involved in mediating leptin's action. The presence of STAT3 binding sites in hTERT promoter and the role of STAT3 in leptin response, suggest that these sites may be involved in leptin's control of hTERT expression. Chromatin immunoprecipitation assays were performed with all putative STAT3 binding sites. In HepG2 cells, STAT3 was found to be associated with site 1 and 2 within hTERT promoter. Short and long term leptin stimulation (200 ng/ml for 48 h and 100 ng/ml for 2 months) of HepG2 led to the recruitment of STAT3 at the hTERT promoter (Figure 3a). In addition, using ChIP analysis we obtained direct evidence for the interaction between c-Myc, Mad1, Max and acetylated H3 with hTERT promoter. In untreated HepG2 cells an hTERT signal was observed in the Mad and Max immunoprecipitations, whereas in leptin treated cells (200 ng/ml for 48 h ) a strong hTERT signal was ditected in the Myc/Max immunoprecipitations (Figure 3b). Interestingly, long term (two months with 100 ng/ml) leptin treatment of HepG2 attenuated the binding of Myc/Max to hTERT promoter. On the other hand acetylated H3 was found to bind on hTERT promoter only after long term leptin treatment (2 months) (Figure 3b).

### Leptin administration affects cell proliferation and modulates the cell cycle of HCC cells

**Figure 4:**
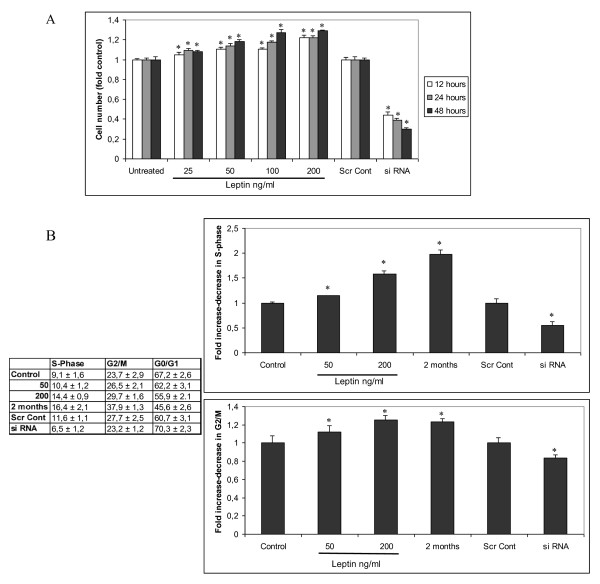
**Leptin is mitogenic for HCC cells**. (a) Effect of leptin on HepG2 cell proliferation. Cell number is expressed as percentage of control, i.e. cell cultures that was untreated. Columns, mean of three independent experiments done in triplicate; bars, SD, *, p < 0.001 compared to untreated cells (for siRNA experiment compared to siRNA control), (b) Leptin icreased the fraction of HepG2 cells in S and G2/M phases of the cycle. HepG2 cells were exposed to leptin (50, 200 ng/ml for 48 h and 100 ng/ml for to 2 months) and leptin siRNA. Columns, mean of three independent experiments done in triplicate; bars, SD, *, p < 0.05 compared to untreated cells (for siRNA experiment compared to siRNA control).

As leptin-mediated overexpression of hTERT might lead to tumorigenic growth and deregulated cell cycle, we investigated, next, the effect of leptin on HepG2 cells proliferation using the MTT assay. Leptin stimulated the growth of HepG2 cells in a time- and dose- dependent manner. Furthermore leptin's knockdown was correlated with a notable reduction in proliferation rate (Figure 4a). Additionally, we observed that treatment with leptin deregulated HepG2 cell cycle, as it increased the proportion of HepG2 in S and G2/M phase, while leptin's knockdown decreased the proportion of HepG2 in S and G2/M phase compared to untreated cells (Figure 4b).

### Leptin could affect tumor progression and invasion dynamics in HCC

**Figure 5:**
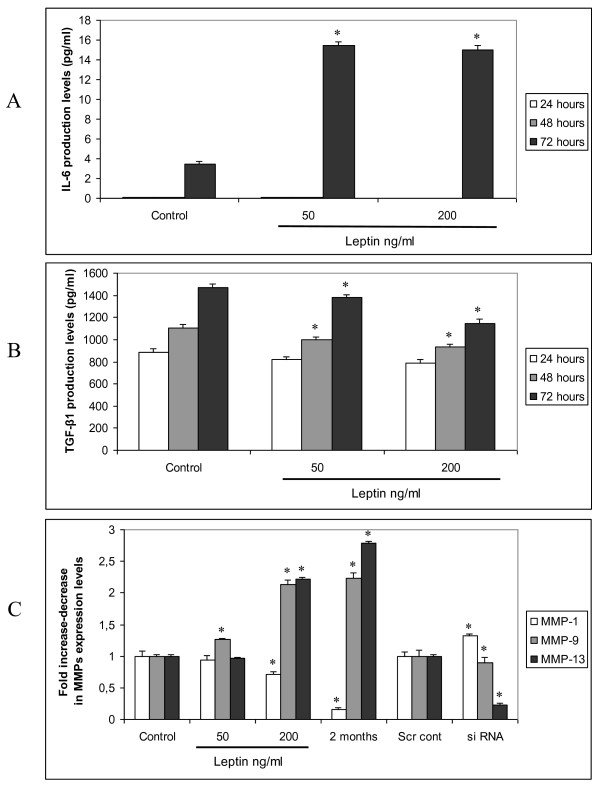
**Leptin regulates inflammatory cytokines and MMPs production in HepG2 cells**. (a, b) Effect of leptin treatment (200 ng/ml) at different times (24, 48, 72 h) on IL-6 and TGF-b1 production. Columns, mean of three independent experiments done in triplicate; bars, SD, *, p < 0.001 compared to untreated cells, (c) Effect of leptin treatment (50, 200 ng/ml for 48 h and 100 ng/ml for 2 months) and leptin siRNA on MMP-1, MMP-9 and MMP-13 protein levels.

The possible role of the inflammatory cytokines in the development and spread of cancer cells led us to examine the involvement of leptin in the production of IL-1a, IL-1b, IL-6 and TGF-β1 by human HCC cells. We found that leptin enhanced only the production of IL-6, after 72 hours treatment and repressed the production of TGF-β1 in a time- and dose dependent manner (Figure 5a, b). Regarding IL-1a, there was no significant difference between stimulated with leptin and untreated HepG2 cultures (data not shown). Leptin siRNA treatment did not affect the production of the above mentioned cytokines (data not shown). As metalloproteinases (MMPs) have been linked with the promotion of tumor invasiveness, we next examined leptin's effect in the production of MMPs-1, -9 and -13 by HepG2 cells. We found that leptin decreased MMP-1 levels and increased MMP-13 and MMP-9 levels in a dose- and time- dependent manner (Figure 5c). siRNA treatment against leptin in HepG2 cells resulted in a significant induction of MMP-1 and reduction of MMP-9 and MMP-13 expression levels (p < 0.001).

### Histone H3 modifications contribute to leptin gene regulation in HCC cells

**Figure 6:**
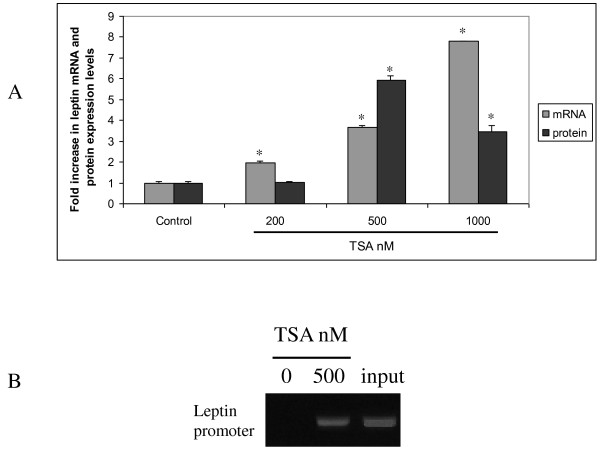
**Histone acetylation regulates leptin expression in HepG2 cells**. (a) TSA treatment (200, 500, 1000 nM) of HepG2 cells for 24 h increased leptin's mRNA and protein expression. Columns, means of three independent experiments done in triplicate; bars, SD, *, p < 0.001 compared to untreated cells, (b) After TSA treatment H3 was found acetylated in proximal leptin promoter.

In order to investigate whether the amount of acetylated H3 interacting with leptin's proximal promoter was correlated with the regulation of leptin gene transcription, we used trichostatin A (TSA), an inhibitor of histone deacetylation. TSA treatment (200, 500, 1000 nM) of HepG2 cells increased leptin's mRNA expression in a dose dependent manner ( ~2, ~3.5, ~8 fold increase in leptin's mRNA expression respectively). The same treatment also upregulated leptin's protein expression, but not in the same pattern (Figure 6a). We tested the acetylation levels of histone H3 and found that in the absence of TSA, H3 binding on the promoter of leptin was undetectable, whereas in TSA treated (500 nM) HepG2 cells, a strong leptin promoter signal was detected in the acetylated H3 immunoprecipitations (Figure 6b).

## Discussion

Numerous studies have established a relationship between obesity and various disease states including cancer. Obesity has been suggested as an important risk factor for both cirrhotic and non-cirrhotic hepatocellular carcinoma, which constitutes the third leading cause of cancer death worldwide [2,26]. It has also been suggested that there is a strong link between leptin and cancer growth and development, with increasing evidence on the involvement of leptin on breast, ovarian, endometrial, colon, and prostate cancer [27-33]. Recently, high leptin and leptin receptor expression levels were correlated with the degree of angiogenesis in human HCC [34]. In addition, leptin-mediated neovascularization showed an effective role of leptin in the development of hepatocarcinogenesis in non-alcoholic steatohepatitis [35]. In the present study, in order to determine the contribution of the leptin system in HCC progression, we investigated the expression of leptin and its receptors in HCC and normal liver tissues. The observed absence of leptin expression in normal liver tissues and it's remarkable presence (78.2%) in HCC liver, accompanied by the elevated OB-Rl and OB-Rs mRNA expression levels in HCC, support the role of leptin system in the development of HCC [36,37]. As the high expression of leptin and its receptors in HCC liver tissues was not found to be correlated with BMI we could assume that the production of leptin in HCC liver is not directly regulated by the adipose tissue deposit, but also reflects the intricate interactions taking place into the tumorigenic microenvironment.

It has previously been reported that hTERT mRNA overexpression and elevation of TA might be some of the processes involved in tumour initiation and progression in the liver [17,23,38]. Our results demonstrate, for the first time to our knowledge, a strong correlation between leptin expression and hTERT levels in HCC liver tissues. Moreover, we found that leptin was capable of a direct beneficent action upon hTERT mRNA and TA in HepG2 cells. The fact that leptin's knockdown by siRNA did not decrease hTERT mRNA levels and TA, suggests that the basal hTERT levels are not only under the control of the leptin system. These findings are in accordance with a very recent study by Ren et al. in MCF-7 cells and reveal that hTERT is probably a target gene for leptin and strengthen the role of leptin as a pivotal factor in HCC [39].

Previous studies have shown that STAT3 is a key mediator of critical cancer cell processes, as it promotes cell cycle progression and survival, stimulates angiogenesis and generally promotes malignant transformation [13,14,40,41]. Very recently, hTERT has been identified as a direct downstream gene of STAT3 in both tumor and normal cells [42]. Taking into account that STAT3 is downstream of leptin and upstream of hTERT, we investigated the hypothesis that the STAT3 signalling pathway plays a crucial role in leptin-mediated hTERT expression. Our findings showed a recruitment of STAT3 in two binding sites in hTERT promoter under leptin stimulation of HCC cells, supporting the key role of STAT3 signaling in leptin induced hTERT expression.

A number of interesting reports have proposed the identification of the Myc/Max/Mad network, as a molecular switch that either interacts with the core promoter to activate hTERT transcription (Myc/Max) or promotes down regulation of hTERT mRNA production (Mad/Max) [43-45]. In the present study we demonstrated, for the first time, an association between the switch from Mad1/Max to Myc/Max binding and activation of hTERT transcription after leptin treatment of HepG2 cells and additionally an expanded interaction of Myc/Max complex accompanied by an increase in H3 acetylation in hTERT proximal promoter after long term leptin treatment of HCC cells. As the long term leptin treatment of HepG2 cells did not extend further the mRNA production of hTERT and TA, we assume that leptin-mediated hTERT overexpression is also under the consistent control of post-transcriptional regulators.

HCC arises most frequently in the setting of chronic liver inflammation and moreover cytokines, such as IL-6, produced in the inflammatory tumor microenvironment stimulate the growth of cancer cells and tumor invasiveness [46]. In the present study, we demonstrated the ability of leptin to increase IL-6 secretion in HCC cells, suggesting that an alternative indirect and independent of the OB-R presence mechanism might be involved in leptin-mediated hTERT expression through JAK/STAT3 pathway. Furthermore, the fact that leptin repressed the production of TGF-b1, a known negative regulator of hTERT [47] represents one more step towards the understanding of the molecular mechanism of leptin action in HCC and the proof of power of leptin-hTERT axis in the tumorigenic processes. To gain insight into the biological effects of leptin's action in the progression and invasion of HCC, we next examined leptin's effect in the production of MMP-1, -9, 13 by hepatocarcinoma cells. Many secreted MMPs are nearly absent in healthy, resting tissues, although they are deregulated in active tissues, as in liver fibrosis and tumor metastasis [48]. In our study we observed, for the first time, that leptin is able to suppress MMP-1 expression and trigger MMP-9 and MMP-13 expression in HepG2 cells, and this could contribute to a more favourable environment for invasion and metastasis of HCC in the cirrhotic liver.

In order to elucidate the signalling cascades in liver cancer, the regulatory mechanisms of genes altered in HCC cells need to be determined. In our study, for the first time to our knowledge, we found that the amount of acetylated H3, in HCC cells, interacting with leptin proximal promoter was correlated with the regulation of leptin gene transcription. The importance of this finding lies in the fact that histone acetylation is reversible and thus may have therapeutic potential.

## Conclusions

In conclusion, our data revealed, for the first time, that leptin up-regulates hTERT expression and TA and deciphered the molecular mechanisms responsible for their interaction in HCC, thus establishing a clearer view of leptin-mediated HCC cell proliferation and progression.

## Competing interests

The authors declare that they have no competing interests.

## Authors' contributions

NS carried out the molecular genetic studies and drafted the manuscript, VP participated in the design of the study and in the molecular genetic studies, YF and YN provided HCC liver tissue samples, AT conceived of the study, and participated in its design and coordination and helped to finalize the manuscript. All authors read and approved the final manuscript.

## Pre-publication history

The pre-publication history for this paper can be accessed here:

http://www.biomedcentral.com/1471-2407/10/442/prepub
